# Correspondence

**Published:** 1869-01-01

**Authors:** G. Newkirk

**Affiliations:** Bradford, Ill.


### *CORRESPONDENCE.*

---

BRADFORD, ILL., *December* 27, 1868.

EDITOR CHICAGO MEDICAL JOURNAL,

*Dear Sir:* My friend, Dr. L. Bevier, of Linn County, Mo., reports to me the following case, which I think worthy of publication. His practice lies in one of those malarious districts so common to that State, and where cases of the so-called congestive chill and pernicious fever of Wood, are, during the summer and fall months, very prevalent.

On the 25th of July, Mr. Lambert, living close at hand, came running in great haste, saying that his son, a lad of sixteen years, was thought to be dying. Upon the Doctor's arrival, a few moments later, the boy was, to all outward appearances, dead. Respiration had entirely ceased, there was no perceptible pulse, and the surface of the body was cold, and covered with a clammy sweat. Artificial respiration was immediately resorted to, and kept up for a space of two hours, before the natural breathing was resumed. Blisters were applied to the legs and arms, and the body continually rubbed with different substances of an irritant nature. As soon as swallowing could be induced, *Tinc. Capsicum* was administered in table-spoonful doses, every fifteen minutes, until he was able to speak, which was not until four hours from the time artificial respiration was commenced. From this on, under the continued use of *Capsicum*, with the addition of brandy and *Quinia*, the lad gradually mended, and within ten days was able to go about.

The case is remarkable for the extreme severity of the onset, and the sudden and powerful action of the active cause; and, also, for the great perseverance used for the measures taken for producing re-action, the chief of which was artificial respiration. It shows us that a want of perseverance in such cases in the use of efficient means, is little better than a crime.

Very respectfully yours,

G. Newkirk, M.D.

